# Thin filament cardiomyopathies: A review of genetics, disease mechanisms, and emerging therapeutics

**DOI:** 10.3389/fcvm.2022.972301

**Published:** 2022-09-07

**Authors:** Lucas K. Keyt, Jason M. Duran, Quan M. Bui, Chao Chen, Michael I. Miyamoto, Jorge Silva Enciso, Jil C. Tardiff, Eric D. Adler

**Affiliations:** ^1^Department of Internal Medicine, University of California, San Diego, San Diego, CA, United States; ^2^Department of Cardiology, University of California, San Diego, San Diego, CA, United States; ^3^Providence Health, Mission Viejo, CA, United States; ^4^Department of Medicine and Biomedical Engineering, University of Arizona, Tucson, AZ, United States

**Keywords:** thin filament, cardiomyopathy, TNNI3, TNNT2, TNNC1, TPM1, ACTC1

## Abstract

All muscle contraction occurs due to the cyclical interaction between sarcomeric thin and thick filament proteins within the myocyte. The thin filament consists of the proteins actin, tropomyosin, Troponin C, Troponin I, and Troponin T. Mutations in these proteins can result in various forms of cardiomyopathy, including hypertrophic, restrictive, and dilated phenotypes and account for as many as 30% of all cases of inherited cardiomyopathy. There is significant evidence that thin filament mutations contribute to dysregulation of Ca^2+^ within the sarcomere and may have a distinct pathomechanism of disease from cardiomyopathy associated with thick filament mutations. A number of distinct clinical findings appear to be correlated with thin-filament mutations: greater degrees of restrictive cardiomyopathy and relatively less left ventricular (LV) hypertrophy and LV outflow tract obstruction than that seen with thick filament mutations, increased morbidity associated with heart failure, increased arrhythmia burden and potentially higher mortality. Most therapies that improve outcomes in heart failure blunt the neurohormonal pathways involved in cardiac remodeling, while most therapies for hypertrophic cardiomyopathy involve use of negative inotropes to reduce LV hypertrophy or septal reduction therapies to reduce LV outflow tract obstruction. None of these therapies directly address the underlying sarcomeric dysfunction associated with thin-filament mutations. With mounting evidence that thin filament cardiomyopathies occur through a distinct mechanism, there is need for therapies targeting the unique, underlying mechanisms tailored for each patient depending on a given mutation.

## Introduction

Cardiomyopathies represent a group of inherited disorders that affect the myocardium with varying phenotypes including hypertrophic cardiomyopathy (HCM), dilated cardiomyopathy (DCM), restrictive cardiomyopathy (RCM), and arrhythmogenic right ventricular cardiomyopathy (1). Genetic mutations can affect any portion of the sarcomere including the thin and thick filaments, titin or calcium handling proteins, ultimately leading to cardiac muscle dysfunction (2, 3). In patients with cardiomyopathies due to sarcomeric mutations, thick filament mutations are the most common and best characterized, but thin filament mutations may account for a clinically significant portion of cases (4–7). Evolution in our understanding of cardiomyopathies and molecular structure of the cardiomyocyte has uncovered many different phenotypic presentations.

Although mutations of the thin filament proteins are a less common cause of inherited cardiomyopathies, it remains clinically relevant as they have distinct clinical presentations and some early studies suggest they are potentially associated with worse outcomes. Morbidity is significant in this subgroup as there are higher rates of restrictive physiology (8), earlier progression to advanced heart failure and higher rates of invasive intervention (9). Although controversial, some early studies have suggested that thin filament cardiomyopathies possibly have a higher rate of mortality, often presenting as sudden cardiac death (SCD) (10), although this remains to be confirmed in larger more contemporary trials.

Treatment has predominantly focused on symptom management related to outflow tract obstruction and heart failure, but it is less clear how to manage patients if these symptoms are not present. Novel targeted therapies currently under development, which address the underlying cause of disease (the dysfunctional sarcomere), may help treat the latter group. Improved understanding of genetype-phenotype relationships in thin filament cardiomyopathy are needed to develop new targeted therapies. The primary purpose of this review is to define the unique epidemiologic, genetic, pathophysiologic, and clinical features of thin filament cardiomyopathies with the intention of improving the recognition and overall management of this rare, but devastating disease subtype.

## Role of the thin filament in the sarcomere

The thin filament is integral to the function of the sarcomere, serving primarily as a path for myosin function. The thin filament is formed through the combination of actin encoded by the gene *ACTC1*, tropomyosin (Tpm) encoded by the gene *TPM1*, and the troponin (Tn) complex, and requires precise coordination of all subunits to function properly. Cardiac actin, composed of two intertwined helical strands of actin requires leiomodin (Lmod), a strong filament nucleator, for nucleation and subsequent polymerization (11). Additionally, tropomodulin (Tmod) binds and stabilizes the minus terminus of actin, effectively end-capping and preventing dissocation of actin monomers (12). Cardiac actin is integrated with Tpm, a dimer of two α-helices wound into a coil that rests in the grooves of actin and binds every seventh successive actin protomer in a spiraling fashion (13, 14). The Tn complex is composed of cardiac troponin-T (cTnT), which associates with Tpm, cardiac troponin-C (cTnC), which contains a calcium binding site, and cardiac troponin-I (cTnI), a regulatory unit inhibiting the binding of actin to myosin. cTnT, cTnC, and cTnI are encoded by genes *TNNT2, TNNC1* and *TNNI3*, respectively.

**Figure 1:**
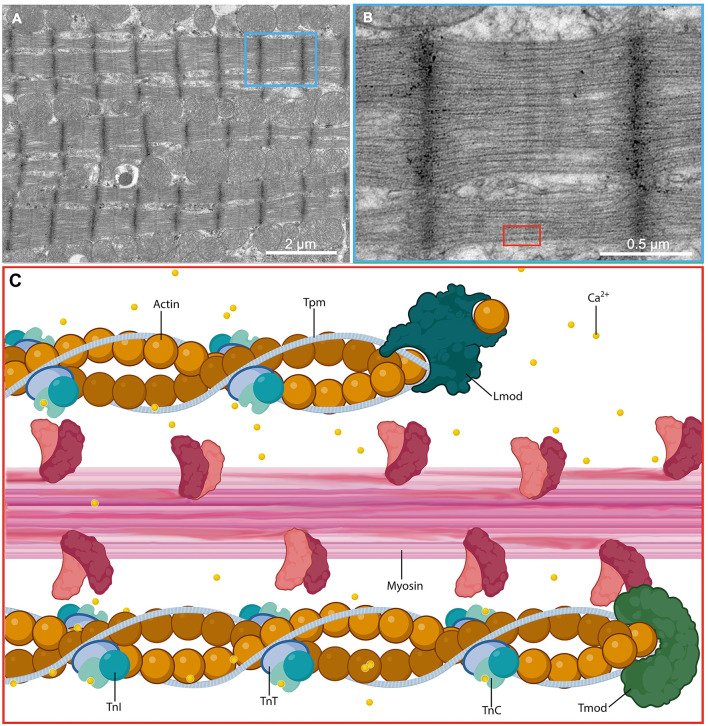
Molecular structure of the cardiac sarcomere. **(A)** Low and **(B)** high magnification electron micrographs showing the sarcomeres of a cardiac myocyte in a glutaraldehyde-fixed heart from an adult male C57BL/6 mouse. **(C)** A magnified schematic illustrating the molecular ultrastructure of the sarcomere and demonstrating the interaction between thick filaments that act as the motor apparatus of the cell and drive contraction and thin filaments that regulate the actions of the thick filaments in response to calcium flux. Ca^2+^, calcium ion; TnC, troponin C; TnI, troponin I; TnT, troponin T.

Ionized calcium plays a key role within the myocyte by coupling electrical stimulation with sarcomeric contraction. Action potentials propagate along the sarcolemma to T-tubules, triggering the influx of calcium through L-type calcium channels into the cardiomyocyte cytoplasm which induces calcium-mediated calcium release from the sarcoplasmic reticulum. Muscle contraction is regulated by changes in intracellular calcium concentration, inducing myosin-dependent changes in the location of Tn and Tpm over the surface of the actin-based thin filament (15, 16). Intracellular calcium concentration is maintained exceptionally low at 1 × 10^−6.5^ M in resting cardiac muscle, during which the N-terminal EF-hand of cTnC is closed and without calcium bound, cTnI is tightly bound to actin and Tpm, and Tpm is stabilized in an inhibitory position, obstructing actin binding sites and preventing myosin from interacting (17, 18). With increased calcium levels in the cytoplasm, calcium bound to cTnC allows the regulatory switch of cTnI to interact with the opened EF-hand of cTnC, removing the inhibition of cTnC on Tpm and actin, exposing the myosin head binding sites on actin. With adequate phosphorylation potential (ΔG-ATP), myosin may bind to actin and initiate cross-bridge cycling, resulting in sarcomeric contraction (Figure 1).

## Occurrence of thin filament cardiomyopathy

Large retrospective studies suggest mutations occurring in *TNNT2, TNNI3, TPM1* and *ACTC* likely account for ~5–15% of all HCM cases, with some reports as high as 30% of HCM cases (4, 5, 19–22). Thin filament mutations can also present as DCM, with *TNNT2* mutations accounting for up to 3% of DCM cases (23–26). RCM has also been associated with thin filament sarcomeric mutations and can present with a severe phenotype with early need for cardiac transplant and premature death (26, 27). Furthermore, the same patient may progress from different phenotypes over the trajectory of their disease. For example, patients may present as a HCM phenotype initially and then develop more of a RCM/DCM phenotype as the disease progresses.

Because these inherited cardiomyopathies have variable penetrance, there is likely a significant proportion of individuals who are carriers, but are asymptomatic without any clinical manifestations (28). Therefore, the overall prevalence of thin filament associated cardiomyopathies may be higher than the estimates provided above.

## Pathologic mutations and clinical presentation

Cardiomyopathies were once regarded as isolated, linear diseases of heart muscle with established clinical presentations and cardiac manifestations that progressed over time. This is consistent with the initial, gross portrayal of HCM, which was a disease of idiopathic subaortic stenosis caused by asymmetric septal hypertrophy (29). With the arrival of molecular analysis and identification of sarcomeric mutations, cardiomyopathies are increasingly recognized as a multifaceted group with significant clinical overlap. Clinical diagnosis of cardiomyopathies can be challenging due to inconsistent presentations and apparent genotype-phenotype incongruity. For example, presentations may be phenotypically distinct (RCM vs. HCM) between family members despite carrying identical mutations (8, 30–32). However, despite the variability of cardiomyopathy presentation there appears to be a number of shared clinical findings observed in patients with thin filament mutations.

Depending highly on the specific amino-acid substitution and associated locus, mutations in each of the thin filament proteins have the potential to manifest as hypertrophic, dilated, and restrictive end phenotypes, with HCM occurring most frequently. Thin filament HCM is most associated with mutations in *TNNI3* and *TNNT2* and may present with “non-classic” findings, including apical hypertrophy, higher risk of SCD, restrictive phenotype, and lesser degrees of left ventricular hypertrophy and LV outflow tract obstruction than that observed with thick filament mutations (5, 9, 33–37).

Several studies have further described clinical manifestations of thin filament HCM through comparison against thick filament HCM. One such study by Coppini et al. suggested increased morbidity associated with thin filament HCM. Specifically, advanced heart failure was observed more frequently with thin-flament mutations than with thick filament mutations, as these subjects had more than twice the likelihood of progressing to NYHA functional class of III or IV, independent of LV outflow obstruction (9). Similarly, LV systolic dysfunction tended to be more severe and occurred at younger ages in patients with thin filament mutations (9), but there are conflicting reports (19).

Thin filament HCM patients may be associated with higher rates of catheter ablation procedures for atrial fibrillation but lower rates of invasive septal reduction therapies, compared to thick filament HCM. There were no differences in ICD implantation rates (9). Dynamic outflow tract obstruction was significantly less common in patients with thin filament HCM, presumably due to the less severe and atypical hypertrophy resulting in less anterior motion of the mitral valve leaflet (9). Diastolic dysfunction was more prominent in patients with thin filament HCM, as suggested by echocardiographic imaging studies (9).

Despite lower rates of hypertrophy and outflow tract obstruction, thin filament cardiomyopathy may convey a worse prognosis compared to its thick filament counterparts. Specifically, the degree of LV hypertrophy does not seem to directly correlate with risk of SCD (38). Patients affected by thin filament HCM have a high risk of SCD with positive family history, non-sustained ventricular arrythmias and abnormal blood pressure response with exercise (9, 33). In a retrospective pedigree analysis of families with HCM-associated *TNNT2* mutations, the overall mortality rate was 34%, but as high as 64% in young males (33). Additionally, the incidence of SCD was remarkably high, occurring in 7 of 22 (32%) subjects with *TNNT2* mutations, likely reflecting a particularly malignant mutation. Conversely, Coppini et al. did not find any significant difference between thin and thick filament HCM regarding SCD, cardiac mortality or all-cause mortality rates during a mean of 4.7 years of follow-up (9). Van Driest et al. similarly found no significant difference between subgroups with regards to SCD incidence (19), suggesting that the arrhythmogenicity seen in thin filament HCM may vary depending on the specific genotype. However, current guidelines do not distinguish between thin filament and thick filament HCM with regards to treatment, including implantable cardioverter-defibrillator (ICD) installation and dysfunction (39).

### TNNT2

Located on chromosome 1q32.1, *TNNT2* encodes cTnT, a 35.9-kDa regulatory protein that combines with cardiac cTnI and cTnC to form the trimeric thin filament Tn complex (40–43). Cardiac TnT specifically functions as the Tpm binding subunit within the Tn complex and regulates striated muscle contraction in response to intracellular calcium levels (40, 41, 44).

The cTnT protein can be divided into two primary subsections: the N-terminal TnT1 (residues 1–181; also referred to as TnT_N_), and TnT2 which is the C-terminal portion (residues 181–289) (45–47). The N-terminus is subject to alternative splicing, yielding multiple isoforms in cardiac tissue (48). TnT1 contains an α-helical tail, which tightly binds and restricts the translocation of Tpm dimers, while TnT2 associates with the Tn complex. The highly charged sequence between residues 112 and 136 is evolutionarily conserved among vertebrates and invertebrates, suggesting this segment is likely crucial to the overall function of cTnT and its anchoring to Tpm and actin (49). Moreover, the TnT1-Tpm region may be important for suppression of cross-bridge cycling during the low intracellular calcium phase of diastole (13). Genetic errors within the TnT1 coding region are typically missense or nonsense mutations, yielding a cTnT protein with altered function rather than a complete loss of function (49). TnT1 is of particular interest, as 65–75% of HCM-associated *TNNT2* mutations occur between residues 80 and 180, with hotspots surrounding residues 92–110 and 160–163 (49–53); the former hotspot appears to be the overlap binding region with Tpm and of clear importance. Mutations in these regions generally demonstrate reduced cooperativity of calcium activation, increased cTnT flexibility and ultimately, reduced interactions between cTnT and Tpm (50, 54). Therefore, it appears a likely central disease mechanism revolves around the altered calcium-dependent affinity of cTnT toward its associated sarcomeric subunits, namely Tpm.

Genetic mutations in *TNNT2* are responsible for up to 15% of all HCM cases (5, 19, 50) and as many as 50% of all thin filament cases (9) (Table 1). Cardiomyopathy caused by *TNNT2* mutations, particularly those altering the interaction with Tpm at residue 92, may portend a worse prognosis than other mutations, as one study of this mutation demonstrated a particularly high rate of sudden death with minimal evidence of hypertrophy (5), although this conclusion has not been validated in any other studies. While *TNNT2* is one of the most studied thin filament genes associated with HCM, the majority of its associated mutations remain inadequately understood. Of the nearly 400 *TNNT2* variations registered in ClinVar, more than one-third remain variants of unknown significance (VUS) and one-quarter demonstrate conflicting characterizations (73).

**Table 1:** Genes associated with thin filament cardiomyopathies and estimated incidences.

**Gene**	**Protein**	**Percent of HCM cases**	**Percent of DCM cases**	**Percent of RCM cases**	**Associated cardiac diseases**
TNNT2	Troponin T	5–15% (5, 9, 19, 50)	3–6% (55–59)	3–8% (60, 61)	HCM, DCM, RCM, LVNC
TNNI3	Troponin I	5% (62)	<1% (63)	3–17%(60, 61, 64)	HCM, DCM, RCM
TNNC1	Troponin C	1% (65, 66)	1% (57, 58, 63)	Unknown	HCM, DCM, RCM
TPM1	Tropomyosin	5% (67–70)	<1–2% (57, 59, 63)	3% (60)	HCM, DCM, RCM, LVNC
ACTC1	Actin	<5% (71)	<1% (57, 72)	8% (61)	HCM, DCM, RCM, LVNC, ASD

HCM, Hypertrophic cardiomyopathy; DCM, Dilated cardiomyopathy; RCM, Restrictive cardiomyopathy; LVNC, Left ventricular non-compaction cardiomyopathy; ASD, Atrial septal defect.

Derived primarily through *in silico* models, *in vitro* protein assays or *in vivo* animal models, the proposed disease mechanism resulting from pathogenic *TNNT2* variants follows a general theme of altered behavior of the carboxyl terminus toward Tpm, with varying degrees of abnormality observed depending on the particular nucleotide substitution. Specifically, HCM-associated mutations, particularly those within the TNT1 region, may alter folding of the Tn tail, potentially leading to abnormal calcium sensitivity and positioning of the Tn-Tpm complex on actin (74).

Interestingly, mutations within *TNNT2* also have the ability to manifest as dilated cardiomyopathy (DCM). Some DCM-causing mutations demonstrate a reduced affinity to calcium, thus increasing the threshold required for contraction and ultimately promoting systolic dysfunction (25). In contrast, using human induced pluripotent stem cells (hiPSCs) to characterize 51 *TNNT2* variants, Pettinato et al. demonstrated increased calcium affinity and increased concentrations of *NPPB*, a marker of cardiac hypertrophy, corresponding to increased thin filament activation (73). Thus, HCM and DCM can both result from altered calcium sensitivity of myosin ATPase regulation, highly dependent on the given nucleotide substitution, either promoting cardiac contraction causing hypertrophy or inhibiting cardiac contraction which may lead to DCM.

### TNNI3

*TNNI3* is the second most common genes associated with thin filament cardiomyopathies after *TNNT2* accounting for ~5% of all cases of HCM (62). *TNNI3* mutations are potentially the most common cause of inherited RCM and are less commonly associated with DCM (75). The prevalence of *TNNI3* mutations vary geographically as *TNNI3* mutations occur more frequently than *TNNT2* in a number of subpopulations in Australia, Singapore, and the Czech Republic (76–80).

*TNNI3* is located on chromosome 19q13.4, encoding cTnI, a ~24-kDa regulatory protein found only in the cardiac sarcomere. Cardiac TnI is generally recognized as the “inhibitory” subunit of the thin filament, promoting cardiac relaxation by restricting actin-myosin cross-bridging during low intracellular calcium concentrations. As intracellular concentrations of calcium rise during systole, the C-terminus of calcium-bound cTnC interacts with cTnI, inducing a conformational change in cTnI. The conformational change of cTnI leads to a reduction in affinity toward actin and ultimately allows for actin-myosin cross-bridge formation (81). Thus, cTnI is an important regulatory protein, moderating cardiac contraction in response to intracellular calcium concentrations.

*TNNI3* contains eight exons and the majority of disease-causing mutations occur in either exon seven or eight, which encode the regions interacting with actin and cTnC, respectively (36, 82). The cTnI peptide can be subdivided into five functional domains: (1) an N-terminal extension only found in cardiac TnI (residues 1–30), (2) the stiff α-helical IT arm which contains critical phosphorylation sites at serine 23/24 for protein kinase A, an N-terminal segment responsible for binding the C-terminal of cTnC during systole (residues 34–71) and a segment responsible for binding cTnT (residues 80–136), (3) the inhibitory domain that binds cTnC and actin-tropomyosin (residues 128–147), (4) the switch domain (also called the “triggering domain”) possessing an flexible α-helix responsible that interacts with a calcium-binding pocket of cTnC (residues 147–163), and (5) the mobile domain at the C-terminus (residues 164–210) (46, 47). The majority of known pathogenic mutations to *TNNI3* affect the inhibitory or mobile domains.

The C-terminal third of cTnI containing the mobile region is the most conserved sequence of the peptide and is responsible for binding and stabilizing actin-Tpm (83, 84). This sequence is likely of significant importance, as deletion of only the final 3–5 residues leads to impaired function (85). Interestingly, it is this highly-conserved C-terminal sequence that houses a disproportionate number of HCM-associated mutations (77, 78, 86, 87). In fact, this nucleotide sequence may have the highest density of pathogenic mutations of any thin filament sequence (86). Mutations Asp190Gly, Arg192His, and Arg204 occurring within this conserved region demonstrate associations with both HCM and RCM phenotypes, suggesting single mutations are capable of multiple and/or overlapping phenotypes (75).

Similarly, the inhibitory region is also a hotspot for both HCM-associated and RCM-associated mutations, suggesting its functional importance (88). Specifically, residue 145 of the inhibitory region interacts with cTnC and Tpm-actin (89–91), and mutations in this location are associated with a highly variable cardiomyopathy penetrance even within the same family (82, 92). Arg145Trp is a particularly pathogenic mutation of the inhibitory region that contributes to a hypertrophic phenotype. It has been studied in detail *via* molecular dynamic analyses and demonstrates dissociated calcium-dependent phosphorylation of cTnI, leading to a reduction in interaction between residue 145 and cTnC (93–95). Similar to several mutations in the C-terminal third, the Arg145Trp has also been found to contribute to RCM phenotypes, further indicating single mutations are capable of translational expression (31).

The function of the switch domain of *TNNI3* is less defined in the literature but is a site of important pathogenic mutations. Ala157Val occurring in the switch region is an interesting missense mutation that can manifest as HCM, RCM, and DCM, even in individuals within the same family (82, 96). A mouse model expressing this mutation recently developed by our lab recapitulates the key restrictive features of this disease in the absence of cardiac hypertrophy, which appear consistent with multiple large family descriptions (82, 97, 98). The mechanism remains largely unclear, but may involve altered binding of cTnC to the switch region of cTnI and subsequent malfunction of the inhibitory region of cTnI (99).

The mutation-hotspots of *TNNI3* suggest the site of mutation is highly important in cTnI-associated cardiomyopathies, as a disproportionate number of disease-causing mutations occur in the highly conserved regions responsible for interaction between sarcomeric subunits. Additionally, the repeated demonstration of identical *TNNI3* mutations manifesting as either HCM or RCM suggests the presence of unknown influencing factors, such as modifier genes or epigenetic influences, highlighting the challenge of predicting clinical manifestation and subsequent treatments based on mutation profile alone.

### TNNC1

*TNNC1* on chromosome 3p21.1 encodes the ~18 kDa protein, cTnC, which serves as a sensor to changes in calcium concentration in the myoplasm. Composed of 161 residues, cTnC has two primary globular domains, a regulatory N-terminal domain (residues 1–86) connected *via* a flexible hydrophobic linker (residues 87–92) to the structural C-terminal domain (residues 93–161) (95). Each globular domain has unique EF-hand binding motifs capable of binding divalent cations (100). Specifically, the C-terminal possess two Calcium/Magnesium binding sites (sites III and IV) while the N-terminal has a single low-affinity calcium binding site (site II) (101). Site II of the N-terminus is chiefly responsible for the reversible binding of calcium throughout the cardiac cycle, hence its recognition as the regulatory domain (102). When bound to calcium cTnC conforms from the “closed” to “open” state and is strongly associated with cTnI, removing the inhibitive effect on Tpm and actin, and allowing for cross-bridge cycling (95).

Interestingly, many of the documented thin filament mutations resulting in HCM involve cTnC in some manner, affecting the calcium sensitivity of cTnC in various capacities. However, pathologic variants of *TNNC1* itself appear less frequently in population studies, with *TNNC1* only attributable in ~1% of HCM cases, if at all (65, 66). In a recent meta-analysis, *TNNC1* conveyed the poorest prognosis of each of the cardiomyopathy-associated troponin genes, with the youngest age of diagnosis and highest rates of death, transplant and ventricular fibrillation (66). Furthermore, *TNNC1* demonstrated the highest rates of *de novo* variants, with ~40% of patients lacking a family history of HCM (65, 66). There appear to be no well-defined variant hotspots within *TNNC1*, with one theory suggesting this is attributable to the poor prognosis of *TNNC1* mutations with fewer probands in the general population (66). Additionally, fewer cardiomyopathy-associated pathogenic mutations have been identified in *TNNC1* compared to other thin filament genes, such as *TNNT2* and *TNNI3*.

The disease mechanism of *TNNC1* pathogenic mutations seem to parallel other thin filament variations with increased sensitivity toward cytoplasmic calcium, leading to amplified contractility during systole and impaired relaxation during diastole (103). A number of mutations, such as Ala31Ser, demonstrated an increased calcium affinity at the N-terminal regulatory binding site II (ΔpCa_50_ = +0.17), promoting contraction at lower calcium concentrations (102). However, other *TNNC1* variations, including as Ala8Val, Cys84Tyr, Leu48Gln, Leu29Gln, and Asp145Glu, result in significantly higher calcium sensitivity abnormalities through mechanisms not involving the N-terminal regulatory binding site, such as stabilizing the most active (M) state of the actin-Tpm-Tn complex and modifying the overall structural dynamics on cTnC (65, 104–106). Interestingly, structural analysis of the Leu29Gln mutation revealed minimal structural alterations to cTnC, but rather abnormal calcium sensitivity and force-generating cross-bridging (107). Therefore, it appears *TNNC1* mutations impair cTnC function through a variety of mechanisms, ultimately increasing calcium sensitivity.

### TPM1

Located on chromosome 15q22.2, *TPM1* contains 14 exons encoding the 32.7 kDa coiled-coil protein, tropomyosin 1.1 (Tpm1.1, also traditionally referred to as α-Tpm), a thin filament protein expressed in both cardiac and fast skeletal muscle fibers (108). Tpm1.1 was one of the first thin filament proteins to be associated with HCM more than 25 years ago (109), with approximately 30 culprit mutations identified since then (67, 110). Furthermore, Tpm1.1 mutations have also been implicated in DCM with associated reduced frequency of actin-myosin interaction. Assessments vary widely regarding the frequency of *TPM1*-associated cardiomyopathy cases, though it is likely low with most estimating *TPM1* is responsible for about 3–5% of all HCM cases (67–70). Interestingly, mutations in *TPM1* result only in cardiomyopathy, with no associated clinical myopathy despite also coding for skeletal muscle protein, possibly due to alternative splicing of the C-terminus (67).

Tpm1.1 can be organized into 7 sections or “periods,” each with an N-terminal α band that interacts with actin and a C-terminal β band which interacts with myosin heads (111, 112). Tpm1.1 wraps longitudinally along the dual grooves of the actin filament, interacting with seven actin monomers and the Tn complex, and forming a regulatory constituent sterically blocking cross-bridge formation in low calcium concentrations. With calcium bound to cTnC, Tpm1.1 is allowed to shift its position, exposing strong cross-bridge binding sites on actin (113).

The α-helices of Tpm1.1 are determined by a strict heptad repeat (*a-b-c-d-e-f-g*)_n_, with hydrophobic residues *a* and *d* internalized within the core and polar residues *e* and *g* facilitating electrostatic interactions with adjacent chains (114). Therefore, the primary structure and sequence of α-tropomyosin is of particular importance, making even single point mutations significant. Unlike some of the other thin filament genes, HCM-associated mutations are distributed throughout the majority of *TPM1* (67). However, a disproportionate number of HCM-associated mutations appear to occur at *g* and *e* positions of the heptapeptide repeat, including Arg21His (*g* position), Ala63Val (*g* position), Asp175Asn (*g* position), and Glu180Gly (*e* position), Glu180Val (*e* position), and Ser215Leu (*e* position) (109, 115–118). Often HCM-associated mutations following this pattern present with significant structural defects such as reduced alpha helix content (119). Furthermore, relatively few HCM-associated mutations have been demonstrated in the hydrophobic *a* and *d* positions, with notable exceptions including Ala22Ser, Ile172Thr, Ile284Val (*d* position) (19, 120, 121). Therefore, it appears changes in the highly-conserved structure of Tpm1.1 may be responsible for pathologic variations in function contributing to cardiomyopathies.

A number of models exist in the literature attempting to describe the mechanism by which *TPM1* mutations lead to HCM (67). One such “overlap” hypothesis is *TPM1* mutations alter Tpm1.1 interactions with the Tn complex, particularly cTnT, likely increasing calcium sensitivity within the myofilament (44, 112, 122). Residues 175–190 are located in exon 5 and are of particular significance, as they correspond to the region responsible for binding cTnT (123). Several mutations within this region exist, with Asp175Asn and Glu180Gly being the most heavily characterized, and may destabilize Tpm1.1-Tn interactions, ultimately causing abnormal cTn complex behavior and altered calcium sensitivity (124, 125). Interestingly, striated muscle tissue biopsies from the vastus lateralis of HCM patients possessing the Asp175Asn mutated α-tropomyosin also demonstrated a similar increase in calcium sensitivity, but no significant change in force conduction or shortening velocity (126). However, this is unlikely to be a unifying theory as dozens of HCM-associated mutations have been reported since the initial characterization of Asp175Asn and Glu180Gly that position outside of residues 175–190.

A second model describes *TPM1* missense mutations leading to weakened interactions between Tpm1.1 and actin, measured as increased free energy. Thus, it is plausible, that weakened Tpm-actin binding would lead to reduced steric inhibition of strong cross-bridge binding sites, loss of Tpm inhibitory function and increased transition into active Tpm states, eventually promoting contraction at lower calcium thresholds (127, 128). This model appears to hold true for mutations that occur at regions responsible for binding actin, for example Ala95Val (*d* position), which is thought to lead to increased binding of Tpm1.1 to actin (67, 123, 129), In summary, there are multiple models that may offer explanation for a particular subset of *TPM1* mutations contributing to the development of HCM.

Mutations in *TPM1* are also moderately associated with DCM, with about thirty identified mutations accounting for 30–35% of familial DCM cases (130, 131). Similar to HCM-associated mutations, those promoting DCM are distributed throughout Tpm1.1 likely are dependent upon positioning within heptad repeat. Mutations Glu40Lys and Glu54Lys (both *e* position), Glu114Gln and Glu62Gln are each reverse local charge from negative to positive and are associated with DCM (130, 132–135). Surface charge reversal (130, 135) appears to affect the stability of the coiled coil superstructure and may reduce affinity toward actin, theoretically leading to lower myofilament tension generation (136).

### ACTC1

Actin is one of the most abundant proteins in human cells, with the most protein-protein interactions of any known protein (137). α-actin, the primary isoform of actin found in cardiac, skeletal, and smooth muscle tissue, is a 42.0 kDa protein composed of 377 residues encoded by *ACTC1* on chromosome 15q14 (40). Due to their respective positions within the overall actin filament, there are two primary domains of α-actin: the outer domain (also referred to as the smaller domain) containing subdomains 1 and 2, and the inner domain (also referred to as the larger domain) containing subdomains 3 and 4 (137).

Mutations in *ACTC1* have been linked with both HCM and DCM, as well as left ventricular non-compaction, with at least 12 mutations associated with HCM (138). Mutations in *ACTC1* account for approximately 1% or fewer of all HCM cases (71). HCM-associated mutations in *ACTC1* tend to primarily congregate in regions responsible for interacting with myosin, Tpm or both (138).

The well-characterized and notably arrhythmogenic mutation, Glu99Lys, was first described in 2000 as familial cardiac hypertrophy (139). Glu99Lys occurs in the myosin-binding region and has demonstrated increased calcium sensitivity with subsequent filament activation in human induced pluripotent stem cell-derived cardiomyocytes likely due to altered binding of myosin (140, 141). Additionally through *in vitro* motility assays, Glu99Lys demonstrated impaired relaxation and fewer motile filaments (142). Two similar mutations promoting HCM, His88Tyr, and Arg95Cys, also occur in the myosin-binding region and have been reported in pediatric populations (143).

Mutations Ala230Val and Arg312Cys, occur in actin regions important for binding Tpm and are associated with HCM (140, 144). Ala230Val produces a missense mutation occurring in the Tpm binding domain, which has demonstrated increased calcium sensitivity and subsequent hypercontraclitlity (140). No change in actin-myosin behavior was noted with Ala230Val, further suggesting it is a mutation affecting only the Tpm binding domain (145). Arg312Cys results in a hypertrophic phenotype, however calcium sensitivity data are lacking (145). HCM-associated mutation Tyr166Cys occurs in a region interacting with both Tpm and myosin, thus altering interactions with both subunits (146). Conflicting evidence exists regarding overall effect on ATPase rate and impact on calcium sensitivity is unknown.

To date, 4 documented mutations in *ACTC1* have been associated with DCM and may account for approximately 1% of DCM-associated mutations (147). Known DCM-causing mutations include Glu361Gly, Thr128Ile, Ile252Met, and Arg312His (72, 147). Interestingly, Arg312His, occurring in the same region as HCM-associated Arg312Cys mentioned above, promotes DCM (72). Despite the dilated phenotype, Arg312His has been shown to result in higher calcium sensitivity with lower calcium concentrations required for myosin activation, suggesting further characterization is required for this mutation along with Arg312Cys (72, 147, 148).

Interestingly, mutations associated with congenital heart defects, namely atrial septal defects, typically occur in the first half of *ACTC1*, while mutations resulting in cardiomyopathies are primarily found in the latter half of the sequence, suggesting *ACTC1* may play a unique role during embryonic development (139–141, 149).

### Additional thin filament proteins

Lmods, are actin-binding filament nucleators that control the length of the thin filament by promoting polymerization of actin. Actin length is highly regulated within myocardiocytes and proper Lmod function is required for maturation of the cardiac sarcomere. Mutations in *LMOD2*, responsible for encoding Leiomodin subtype 2, have been associated with DCM. The prevalence of *LMOD-*associated DCM is unclear, though likely exceptionally rare in part due to the seemingly high mortality rate. This was demonstrated clearly in *LMOD2* knockout mice, that developed rapid-onset DCM with abnormally short thin-filaments and disorganized myofibrils (150). This was further exhibited through exome sequencing of a neonate with severe DCM (z score range 6.29–2.8), which revealed homozygous non-sense mutation Trp398^*^, interited from both asymptomatic heterozygous parents. The patient required prompt LVAD placement followed by heart transplant at 10 months (151). A similar case of neonatal DCM was demonstrated with biallelic *LMOD2* mutations: Leu415Val causing frameshift and Arg513^*^ as non-sense. Interestingly, it appears heterozygous carriers of *LMOD2* mutations are often asymptomatic, suggesting only a low level of Leiomodin is required for normal function.

Tmod is an actin filament end-capping protein, responsible for stabilization of the pointed end of actin polymers from spontaneous disassociation. Though detailed structural information is somewhat lacking, *in vitro* models suggest it interacts with three actin subunits at the pointed end and also interacts with two Tpm subunits (12). Overexpression of *TMOD1*, encoding Tmod subtype 1 found in cardiac muscle, in mouse models demonstrated shorter thin filaments associated with a DCM phenotype and relatively high mortality rate, not unlike that seen with *LMOD2* mutations (150). Conversely, *in vitro* inhibition of Tmod *via* anti-Tmod antibody resulted in actin elongation from the pointed end (152). In both Lmod and Tmod loss of function studies, there does not appear to be any clear association with abnormal calcium sensitivity, unlike other thin filament mutations, which may indicate an alternative mechanism of cardiomyopathy that may be more dependent upon abnormal sarcomere structure.

## The need for targeted therapies for thin filament cardiomyopathy

Thin filament cardiomyopathies are heterogeneous, with stark differences between seemingly similar mutations involving the same allele. Much remains unknown about the intermediary processes between the inciting mutation and subsequent cardiomyopathy, though there are several themes that remain consistent across all thin filament pathologic variants that ultimately distinguish this subtype of cardiomyopathy.

Common to each of the thin filament pathogenic variants previously discussed, a single nucleotide is typically replaced in a critical and/or highly conserved region of the genetic sequence, which encodes the substitution of a new, single amino acid and likely alters the higher global structure. Often, this occurs in a region responsible for the direct interface with another protein component of the sarcomere, distorting the normal cooperativity of these subunits, or in a key regulatory domain, changing the normal behavior of the protein itself. There is overwhelming evidence these mutations ultimately contribute to dysregulation of calcium within the sarcomere, as opposed to the leading hypothesis of increased ATPase activity observed with increased disordered relaxed state (DRX) of myosin in thick filament HCM (153–155). It is understandable how the theoretical increase in ATPase activity of thick filament HCM would directly promote increased contractility, oxygen consumption, and hypertrophy; however, the pathophysiologic mechanism is less clear in thin filament mutations. While there is increased oxygen and energy consumption with thin filament mutations, ATPase activity is conversely decreased, and myosin adopts the super relaxed state (SRX). This further suggests thin filament mutations promote cardiomyopathy through a separate mechanism compared to those of thick filament mutations (156, 157).

Though there is clinical overlap between subtypes and a high degree of variability at the individual patient level, a number of findings in thin filament HCM appear to be more correlated with thin filament mutations, including younger age of symptom onset, reduced LV hypertrophy with atypical distribution, reduced obstruction, increased diastolic dysfunction, increased progression to heart failure, elevated arrhythmogenicity, and potentially higher mortality (depending on the specific mutation) (9, 33). Additionally, mutations of the thin filament proteins seem to also correlate more with restrictive phenotypes and diastolic dysfunction more so than thick filament mutations (9). These distinctions are likely reflective of the different underlying pathophysiologic mechanisms driving each subtype of cardiomyopathy.

Current management of thin filament cardiomyopathies primarily consists of identification of disease through familial or genetic screening, control of symptoms through medication and lifestyle modification, and mitigation of adverse events such as sudden cardiac death. As recommended by current guidelines, early identification and characterization through genetic testing is paramount for at-risk individuals, such as those with family members diagnosed with HCM (39). However, with increasing use of high-sensitivity genetic testing there is a constantly expanding list of variants of unknown significance, often contributing more to patient confusion and undue distress rather than clinical intervention. As a potential solution, Mason et al. have demonstrated the use of a high-fidelity computational model of the cardiac thin filament to predict point mutation behavior at the atomic level, suggesting the future possibility of characterization of a given VUS without necessitating animal models or familial studies (158). But until mutation characterization matches available screening diagnostics, clinicians should be encouraged to limit any interpretation of genetic testing to only well-understood variants.

In patients with a primarily obstructive phenotype, though less common in thin filament cardiomyopathies, pharmaceutical agents with negative inotropic and chronotropic effects are recommended. Beta blockers are typically the first choice in such patients, as they are especially effective against exercise-induced obstruction and generally well-tolerated (39). However, if beta blockers are not tolerated or do not provide benefit, non-dihyropyridine calcium channel blockers may be trialed instead (39). The class Ia antiarrhythmic, disopyramide, has sarcomere-independent negative inotropic effects and is effective in reducing both obstruction and arrhythmic activity (159, 160). Furthermore, the combination of disopyramide with a beta blocker or non-dihyropyridine calcium channel blocker is likely one of the most effective symptom management strategies for patients with obstructive disease, with more than half of patients experiencing reduced resting outflow tract gradients and reduction in limiting symptoms (161, 162).

In patients with primarily obstructive disease with hypertrophic phenotype who fail medical management, septal reduction therapies, including alcohol septal ablation and septal myectomy, is an option with the intention of prolonging life and/or relieving symptoms. However, surgery is generally recommended only after failure of medical therapy and is infrequently performed in patients with thin filament cardiomyopathy, given the low prevalence of outflow tract obstruction in this population (161).

ICDs are life-saving devices that should be considered in individuals with high-risk features concerning for SCD. In general, ICD is recommended in individuals with HCM and previous cardiac arrest, sustained ventricular tachycardia, sudden death in a first-degree family member, LV hypertrophy >30 mm in any LV segment, a history of syncope due to arrhythmia, LV apical aneurysm or LV systolic dysfunction (39). A number of risk-stratifying tools have been developed to identify patients at risk of SCD, with the enhanced ACC/AHA clinical risk factor strategy reporting a sensitivity for predicting SCD events of 85–95% (163). However, it should be noted that risk-stratifying tools heavily weigh outflow tract obstruction, which is less prevalent in thin filament cardiomyopathies despite an equal or greater rate of SCD. Therefore, these tools should be used with knowledge of potential underestimation of SCD risk in those with thin filament cardiomyopathies.

In HCM, genetic mutations facilitate interaction between myosin heads and actin, and increase duration of myosin in the attached state, ultimately leading to increased force generation, increased contractility and impaired relaxation. Mavacamten (MYK-461) and CK-274, novel reversible allosteric inhibitors of cardiac myosin ATPase, specifically target myosin and reduce actin-myosin cross-bridge formation, limiting contraction and cardiac thickening in a dose-dependent manner (164). In one particular myosin mutation (R403Q) mouse model, mavacamten demonstrated the ability to reduce ventricular hypertrophy if administered early in the disease process (165). A phase 2 clinical trial, PIONEER-HCM, demonstrated a reduction in the post-exercise LVOT gradient, increased exercise capacity and improved symptoms in patients with hypertrophic cardiomyopathy treated with mavacamten (166). Most recently, EXPLORER-HCM, a phase 3 trial involving 251 patients with obstructive HCM treated with either mavacamten or placebo for 30 days, demonstrated significant improvements in peak oxygen consumption, New York Heart Association (NYHA) class, post-exercise LVOT gradient and patient-reported symptom scores (167). Most interestingly, mavacamten appeared to rescue a number of effects associated with the thin filament mutations R92Q cTnT and R145G cTnI as demonstrated through *in vitro* cellular models. Specifically, mavacamten has been shown to reduce cytoplasmic calcium concentrations to below wild-type levels, suggesting mavacamten may also have the capacity to attenuate the heightened calcium sensitivity driving the majority of thin filament cardiomyopathies (168). Thus, it is possible the therapeutic efficacy of mavacamten will vary depending on the specific mutation and underlying disease mechanism. In theory mavacamten would be more likely to affect thick filament mutations with hypertrophy, however, genotypes were not clearly defined in EXPLORER-HCM and their future role or benefit in thin filament mutations, particularly those without hypertrophy or with a predominantly restrictive phenotype is unknown. Sarcomeric modulators, with cardiac myosin ATPase inhibitors being the novel example, certainly show early promise and may represent a landmark in the treatment of HCM, though long-term data involving diverse patient populations and potential drug-drug interactions are still needed (169).

Future therapies may target key alterations in proteins associated with calcium dysregulation observed extensively in thin filament cardiomyopathies. Using two cTnT mouse models with R92L and R92W mutations Lehman et al. demonstrated a significant increase in phosphorylation and subsequent auto-activation of calmodulin kinase II (CaMKII), a protein importantly involved in normal calcium homeostasis within the sarcomere, which was associated with a HCM phenotype (170). Most notably, inhibition of CaMKII lead to partial reversal of this phenotype in one of the models, with improved diastolic function and reduced atrial remodeling, suggesting a potential target for future therapeutics.

Altered calcium sensitivity has also been a focus for therapeutics in the treatment of acute systolic dysfunction. Levosimendan, a pyridazinone-dinitrile derivative and inodilator, was initially developed for acute treatment of decompensated heart failure and functions through multiple mechanisms, of which include increasing calcium sensitization of cTnC and ultimately decreasing the threshold for sarcomere contraction. The ALARM-HF trial demonstrated improved in-patient outcomes for patients with heart failure of various etiologies in acute decompensation (171). In both pediatric and adult patients with severe DCM, repetitive levosimendan infusions have demonstrated improvement in heart failure symptoms, hospilization rates and overall survival while in acute decompensation, however data are limited regarding longterm use with the longer published treatment duration measuring on the order of months (169, 172, 173). In theory, the heightening of calcium sensitization of the sarcomere would be an effective means to offset the lowered calcium sensitivity observed in DCM though extended use is likely limited to select patients with advanced heart failure rather than as a preventative measure in DCM.

With a greater understanding of the genetic mechanisms contributing to cardiomyopathy, there is increased effort to correct pathologic genetic variants prior to their clinical manifestation in patients. Unlike many genetic diseases, thin filament cardiomyopathies are caused by only a few errors in key nucleotide sequences, making them relatively approachable targets for genetic editing. While most gene therapeutic investigations focus on the treatment of thick filament cardiomyopathies, the pathologic mechanisms are similar enough in thin filament cardiomyopathies that much can be expected to translate.

Gene therapy, following the increased accessibility and utility of next-generation sequencing, allows for revision of erroneous genetic material and is often seen as one of the forefront therapies for inborn genetic diseases. One of the key challenges with gene therapy, however, is the transport of correctional material into the affected cells. Several delivery strategies have been used in cardiovascular diseases and offer potential to be used in thin filament cardiomyopathies, including the use of adeno-associated virus-9 (AAV-9), adenovirus, and lentivirus as vehicles for the introduction of genetic material (174–177).

In a proof-of-concept study focusing on loss-of-function hypertrophic mutation, *in vivo* gene transfer of *MYBPC3* encoding cardiac myosin-binding protein-C (cMyBPC) was introduced *via* lentiviral vector-mediated transfer into cMyBPC-deficient mouse myocardium (178). Supplementation of functional *MYBPC3* resulted in near wild-type levels of cMyBPC with improved *in vivo* cardiac function, implying the possibility of delaying the clinical presentation of hypertrophy or circumventing the phenotype altogether. Alternatively, viral vectors have been used to deliver antisense oligoribonucleotides to deactivate gain-of-function mutations in *MYBPC3* through either exon skipping to mask enhancer sequences and subsequently remove a pathologic sequence without inducing a frame shift (179, 180), or trans-splicing, which splices engineered wildtype pre-trans-mRNA with mutant pre-mRNA and yields functional full-length mRNA (181–183). Limitations to these techniques include the potential for inadvertent mutagenesis and oncogenesis through inappropriate insertion, inconsistent or inefficient transduction throughout the myocardium (especially with trans-splicing) and possible host immune response toward viral vectors, such as adenovirus vectors (184).

Gene transfer *via* the cardiotropic adeno-associated virus 9 (AAV9) has demonstrated return toward baseline regulation of intracellular calcium, which may allow for therapeutic adjustment of calcium sensitivity altered in thin filament cardiomyopathies. The sarcoplasmic reticulum calcium-ATPase 2a (SERCA2a) is responsible for the rapid sequestration of cytosolic calcium into the sarcoplasmic reticulum after signal transduction in the myocardium. SERCA2a is inhibited by phospholamban in the unphosphorylated state, a micropeptide protein encoded by *PLN*. Gene transfer of *serca2a* using first-generation type 5 recombinant human adenovirus vector (AV-5) into neonatal mouse myocardium containing a hypertrophy-inducing mutation of thin filament protein tropomyosin demonstrated delayed hypertrophy and improved cardiac function at 6 weeks (185). SERCA2a delivery using a separate vehicle, adeno-associated viral vector, is determined to be a safe therapy in humans in a recent series of clinical trials focusing on chronic heart failure, the most recent of these being AGENT-HF (186–188). This trial failed to show any clinical improvement, though underpowered (186). Similar conclusions were drawn in the CUPID 2 trial, which demonstrated effective AAV1/SERCA2a gene transfer in heart failure patients, though ultimately no survival benefit (189). Interestingly, intracoronary gene transfer of adenylyl cyclase, a 130-kD membrane protein associated with increased SERCA2a calcium uptake, using adenovirus vector was shown to not only be safe but also demonstrated a dose-response improvement in cardiac function in patients with symptomatic heart failure and reduced ejection fraction (190). Although similar effects have been demonstrated through the deletion of phospholamban, leading to increased activity of SERCA2a, other deletions have yielded dilated cardiomyopathy in humans and likely prevent it from being used for treatment of HCM (191, 192).

The use of small interfering RNA (siRNA) cassettes is an alternative method to knock down the expression of pathologic missense mutations. A number of vehicles exist for the trafficking of siRNA, including lipid-based, cell-penetrating peptides, polymers and viral carriers (193). In one particular study, adeno-associated viral vectors were used to deliver siRNA into mouse models hemizygous for wildtype *MYH6* gene to silence the mutant allele, reducing pathologic transcripts by 25% and allowing for adequate production and function of alpha myosin heavy chain to prevent hypertrophy and fibrosis at 6 months (194).

Intracellular introduction of therapeutic material including low molecular weight drugs, proteins, peptides and genetic material including siRNA, and more recently mRNA, *via* lipid nanopartciles (LNP) represents a promising alternative delivery system absent of certain distinguishing drawbacks of viral vectors (195, 196). A number of theoretical advantages are offered by LNPs in the treatment of cardiomyopathies, including possible reduced immunogenicity and high carrying capacity compared to well-established viral vectors (197). However, specifically targeting myocardial tissues with LNPs will likely require further development in order for therapeutic use in cardiomyopathies, as constrated by the innate cardiac honing of AAV9 (198). Early findings from *in-vivo* animal studies demonstrate successful targeted delivery of mRNA to infarcted myocardium, suggesting a similar approach may be feasible in treatment of inherited cardiomyopathies (199).

CRISPR/Cas9 (clustered regularly interspaced short palindromic repeats/clustered regularly interspaced short palindromic repeat—associated 9) technology has been used *ex vivo* to edit sperm cells containing a pathologic *MYBPC3* mutation associated with thick filament HCM. By introducing DNA nicks at regions adjacent to the targeted sequence and provided an engineered template, the pathologic sequence can be replaced through homologous recombination, resulting in correction of *MYBPC3* alleles in 72% of embryos (200). This therapy is purely experimental with the need to prevent adverse events, such as off-target insertions or deletions, and faces clear ethical concerns. Most recently NTLA-2001, a gene-editing therapy using CRISPR/Cas9, has been used for *in vivo* treatment of transthyretin amyloidosis associated with cardiomyopathy and heart failure. Among 6 patients undering going 28 days of NTLA-2001 infusion therapy, a dose-related reduction in misfolded transthyretin was demonstrated with minimal adverse effects. Though further evaluation is required, this is the first *in vivo* demonstration of the ability to eliminate mutations associated with cardiomyopathy (196, 197).

## Conclusion

As screening methods improve and the progression of cardiomyopathy treatment advances from symptom management to preventive and curative therapies, it is paramount to recognize thin filament cardiomyopathies as distinct diseases with a unique pathophysiologic mechanism and clinical presentation.

## Author contributions

LK, JD, and EA contributed to the conception and design of the review article. All authors were involved in the drafting of the manuscript or revising it critically for important intellectual content and approved the final manuscript prior to submission.

## Conflict of interest

JD reports personal fees from Lexeo during the conduct of the review. EA reports personal fees from Abiomed, Novartis, Abbott, non-financial support from Astra Zeneca, personal fees from Ionis Pharmaceuticals, Sana Biotechnology, Medtronic, other from Rocket Pharmaceuticals, Papillon Therapeutics, ResQ Pharmaceuticals, personal fees from Lexeo Pharmaceuticals, Cytokinetics, Endotronics, and during the conduct of the review. MM was employed by Providence Health. The remaining authors declare that the research was conducted in the absence of any commercial or financial relationships that could be construed as a potential conflict of interest.

## Publisher's note

All claims expressed in this article are solely those of the authors and do not necessarily represent those of their affiliated organizations, or those of the publisher, the editors and the reviewers. Any product that may be evaluated in this article, or claim that may be made by its manufacturer, is not guaranteed or endorsed by the publisher.
